# Hormone-switching islet cells: parallels to transmitter-switching neurons

**DOI:** 10.3389/fcell.2025.1587893

**Published:** 2025-04-28

**Authors:** Yuval Dor, Nicholas C. Spitzer

**Affiliations:** ^1^ Department of Developmental Biology and Cancer Research, The Institute for Medical Research Israel-Canada, The Hebrew University-Hadassah Medical School, Jerusalem, Israel; ^2^ Neurobiology Department, School of Biological Sciences and Center for Neural Circuits and Behavior, University of California San Diego, La Jolla, CA, United States

**Keywords:** neurotransmitter switching, islets, hormone-positive, pancreas, plasticity

## Abstract

Although originating from different germ layers, pancreatic islet cells and neurons share extensive similarities, both physiological (e.g., voltage-dependent release of a bioactive molecule) and molecular (e.g., highly similar composition of transcription factors and structural genes). Here we propose that two seemingly unrelated phenomena recognized in these cell types—neurotransmitter switching in neurons and the expression of two or more hormones in individual islet cells—share a deep resemblance, potentially reflecting an ancient molecular circuit of cell plasticity. Comparing and contrasting dynamic hormone expression in islet cells and transmitter switching in neurons may provide insights into the functions and underlying mechanisms of these phenomena.

## Multi-hormonal cells and hormone switching in pancreatic islets

A key characteristic of pancreatic islet cells is the expression of one of several hormones (insulin, glucagon, somatostatin, or pancreatic polypeptide, and, with lower frequency, ghrelin and gastrin), which defines their functional identity (Rieck et al., 2012; Walker et al., 2021). It has been known for many years that during early development, islet cells often co-express several hormones. Although originally considered to be progenitors for later mono-hormonal cells, elegant lineage tracing experiments showed that most embryonic bi-hormonal cells are eventually eliminated and that adult islet cells originate in fetal NeuroG3+ endocrine progenitor cells that differentiate into mono-hormonal cells (Herrera, 2000; Gu et al., 2002). However, a recent study suggested that embryonic islet cells may switch the hormone type that they produce; for example, glucagon+ cells in the embryo may switch to insulin expression in adult life (Perez-Frances et al., 2022). Multi-hormonal cells also appear in embryonic stem cell differentiation protocols aimed at generating beta cells, but they are regarded as a developmental dead end to be avoided in directed differentiation (Pagliuca et al., 2014; Rezania et al., 2014; Bruin et al., 2014; Veres et al., 2019). Overall, we do not yet fully understand how such transient cells are formed at the molecular level, what their evolutionary origin is, and what function they serve, if any.

A related phenomenon, likely more relevant physiologically, is observed when adult islet cells are exposed to metabolic stress. This sometimes results in ectopic expression of hormone genes, with or without the downregulation of the original, identity-defining hormone. It was proposed that such a process taking place in insulin-producing beta cells may contribute to beta cell failure in diabetes (Talchai et al., 2012) and that in the reverse direction, alpha or delta cells may respond to stress by acquiring beta cell identity and turning on the expression of insulin, thus driving beta cell regeneration (Chera et al., 2014; Thorel et al., 2010). Note that we are referring here to hormone expression at the level of individual islet cells; population-wide changes that alter the proportion of cell types in islets—such as beta cell hyperplasia following the consumption of a high-fat diet in mice or alpha cell hyperplasia when glucagon receptor signaling is blocked—represent a different phenomenon, achieved via the proliferation of normal mono-hormonal cells that retain their molecular identity.

The molecular basis of islet cell plasticity has started to emerge in recent years. Although islet cell identity appears to be a stable trait (i.e., islet cells will never take on ductal or exocrine cell identity) (Magenheim et al., 2023), the particular choice of the hormone expressed (defining an islet cell type) is considerably plastic. Studies of key transcription factors (TFs) in islets revealed that many such TFs act as activators of a transcriptional program of a given islet cell type while repressing the expression of alternative islet cell programs. When such TFs are inactivated, the primary identity of an islet cell is weakened, and alternative programs are de-repressed. For example, when the *Pax6* gene is disrupted in beta cells, they turn on the expression of the hormones ghrelin, glucagon, and somatostatin (Swisa et al., 2017a); when Pdx1 is disrupted in beta cells or activated in alpha cells, glucagon or insulin expression is induced (Yang et al., 2011; Gao et al., 2014); when Foxo1 is disrupted, beta cells turn on the expression of multiple hormones (Talchai et al., 2012); and when Nkx6.1 is disrupted in beta cells, they acquire features of somatostatin+ delta cells (Taylor et al., 2013). While most experiments along these lines have used genetically engineered gene disruption, it has also been proposed that some islet TFs, particularly those responsible for maintaining beta cell identity, are sensitive to hyperglycemia-induced oxidative stress, potentially contributing to the loss of beta cell identity in type 2 diabetes (Swisa et al., 2017b; Guo et al., 2013). Thus, general islet cell identity is stable, but the maintenance of a particular cell identity requires active transcriptional maintenance and is susceptible to stress. The biological significance of this arrangement is not clear, and the phenomenon is often observed as an indication of a failed cellular state. Finally, a potentially related phenomenon is hormone-negative islet cells. Such cells often appear in the context of islet cell tumors; they retain the expression of many molecular hallmarks of islet cells but express no hormones (Sobol et al., 1989). The cellular origins of such cells and the underlying mechanism are not known.

## Similarity between islet cells and neurons

Neurons and pancreatic islet cells share deep molecular and functional similarities, although they originate in different germ layers (ectoderm and endoderm, respectively), as shown originally by classical chick-quail experiments (Le Douarin, 1988). Both cell types are typically post-mitotic and long-lived. Both are characterized by the release of a signaling molecule through a process involving altered membrane potential, calcium entry, and fusion of secretory granules with the plasma membrane. The nature of the secreted molecule is obviously different—islet hormones are gene-encoded peptides released into the blood, while neurotransmitters are typically small molecules acting short-range through synapses. Nonetheless, there is a clear, extensive similarity between islet cells and neurons, resulting from their highly similar transcriptomes, including key transcription factors and structural genes functioning in the secretion process. The similarity between neurons and islet cells extends to pathologies. For example, islet amyloid polypeptide (IAPP), a hormone co-secreted with insulin from beta cells, shares structural similarity with amyloid beta, which is co-secreted with neurotransmitters from neurons. Oligomers of these proteins are proposed to contribute to the development of type 2 diabetes and Alzheimer’s disease, respectively (Westermark et al., 2011).

At a more abstract level, islet cells and neurons both generate behaviors: islet cells affect the behaviors of internal organs, and neurons affect behaviors that are evident outside the body. In this study, we draw attention to the retention by both neurons and islet cells of a unique type of plasticity, allowing for a switch of identity even without cell division or differentiation from stem cells. We argue that different aspects of this process are observed in neurons and islet cells and that elucidating their fundamental similarities may provide valuable insights for both fields of research.

## Neurotransmitter switching

Like islet cells, neurons of a given type are specified to produce and release a specific neurotransmitter, which defines their identity and function. Importantly, neurons can sometimes change the neurotransmitter that they produce, a phenomenon termed neurotransmitter switching. This has profound consequences on brain function and plasticity. For example, transmitter switching has beneficial effects on learning motor skills (Li and Spitzer, 2020), modulating social preference (Dulcis et al., 2017), and generating camouflage behavior (Dulcis and Spitzer, 2008) and detrimental effects, including depression (Dulcis et al., 2013), cognitive deficits (Pratelli et al., 2024), and generalized fear (Li et al., 2024). The triggers for neurotransmitter switching are typically external cues, such as light exposure or altered sensory input, and they may bring about a long-term, stable change in cell identity.

**FIGURE 1:**
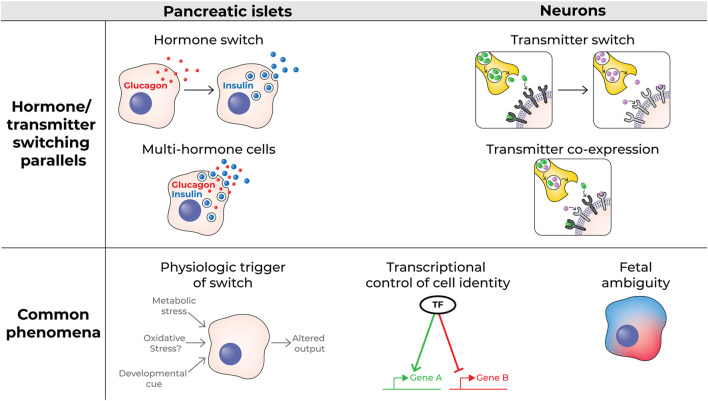
Schematic of hormone switching and multi-hormone cells vs transmitter switching and transmitter co-expressing neurons (top); some characteristics common to both processes (bottom).

The molecular mechanisms underlying neurotransmitter switching are not fully understood, but evidence suggests that sustained changes in neuronal activity play a role (Li et al., 2020). Like double-hormone positive fetal islet cells, embryonic neurons may have a “mixed” phenotype, producing more than one transmitter, and they may later switch (Root et al., 2008; Pereira et al., 2015; Kotak et al., 1998). Neurotransmitter switching involves key transcription factors such as Lmx1b, Nkx2.2 (Demarque and Spitzer, 2010), Tlx3 (Guemez-Gamboa et al., 2014; Marek et al., 2010), Bcl11b, and Pax6 (Dulcis et al., 2017); notably, several of these factors are also known players in islet cell biology and determining the hormone produced by a cell, e.g., Pax6 (Swisa et al., 2017a), Nkx2.2 (Gutiérrez et al., 2017), and Lmx1B (Schreiber et al., 2021). Adult post-mitotic neurons require active maintenance of their identity, including the transmitter they produce, through a transcriptional program (Deneris and Hobert, 2014). Interestingly, transmitter switching may change the valence, e.g., change the product of a cell from excitatory to inhibitory; this resembles a switch in hormone production in an islet cell that flips the metabolic impact of a cell, e.g., from a glucagon-positive alpha cell charged with increasing blood glucose levels to an insulin-positive beta cell that acts to reduce blood glucose levels. Table 1 and Figure 1 highlight some key features of multi-hormonal or hormone-switching islet cells and neurotransmitter switching and point out common themes.

**TABLE 1:** Features of multi-hormonal and hormone-switching islet cells and neurotransmitter switching.

	Pancreas	Brain
Cell type	Islet cells	Neurons
Key product	Hormones	Neurotransmitters
Stability of phenomenon	Requires active transcription	Requires active transcription
Switch phenomenon	Bi/multi-hormonal cells, transdifferentiation	Neurotransmitter coexpression and neurotransmitter switching
Stimulus	Developmental cues, glycemic stress, oxidative stress, and beta cell ablation	Sustained change in electrical activity and environmental stimuli (e.g., stress or drugs)
Result of switch	Reprogramming of islet cell identity to another mono-hormonal islet cell type, a multi-hormonal state, or a hormone-negative state	Transmitter switching often reverses the sign of the synapse: an excitatory transmitter is replaced by an inhibitory transmitter or vice versa
Physiological significance	Naturally occurring multihormonal cells typically considered to represent a developmental error. Hormone switching potentially a mechanism of natural regeneration	Enhanced motor skills, social preference, camouflage, depression, generalized fear, cognitive deficits, and autistic-like behavior
Molecular mechanism	Likely transcriptional derepression of hormones due to the downregulation of key transcription factors that also act as repressors	Transcriptional, at least in part; involvement of Lmx1b, xTlx3, bcl11b, and Pax6
Effect of age	Multi-hormonal cells form in early development; evidence suggests that delta cells may reprogram to beta cells in early postnatal life, while alpha cells may reprogram in adult life	Reduced with advanced age

## Lessons for islet cell biology and neurobiology

Neurotransmitter switching is an active, regulated response of neurons to altered environments, leading to changes in behavior that can be beneficial (Li and Spitzer, 2020) or detrimental (Li and Spitzer, 2020; Dulcis et al., 2013; Pratelli et al., 2024; Li et al., 2024; Godavarthi et al., 2024). In contrast, the altered hormonal identity of specific islet cells (i.e., a cell autonomous phenomenon) is typically regarded as a non-physiological process representing developmental errors or a pathological process contributing to the disruption of glucose metabolism and diabetes. One exception is the proposal that near-total loss of beta cells may trigger a switch in the molecular identity of alpha or delta cells, leading to the partial regeneration of beta cell mass through alpha or delta cell reprogramming (Chera et al., 2014; Thorel et al., 2010). Based on knowledge of neurotransmitter switching, we hypothesize that additional environmental triggers may reveal novel physiological aspects of plastic islet cell identity; for example, a switch in diet composition could lead to the adaptive alteration of hormone expression at the single-cell level. In other words, neurotransmitter switching should inspire islet biologists to search for biological contexts in which the appearance of multi-hormonal cells or hormone switching is an adaptive, regulated response.

On the other hand, the transcriptional regulation of islet hormone expression and repression appears to be better established than the understanding of the transcriptional program of neurotransmitter switching. We hypothesize that, similar to the situation in islets, key neuronal transcription factors (some also expressed in islet cells) act as both gene activators and repressors, controlling the decision of individual neurons regarding which transmitters are produced and which are silenced. Furthermore, islet studies point to transcription factors as sensors of oxidative stress caused by excessive glucose metabolism; a similar process may be at play in neurons that switch transmitters. Studies of gene expression and chromatin accessibility using single-cell or single-nucleus RNA sequencing and single-cell ATAC-sequencing in switching islet cells and switching neurons could be productive and mutually informative regarding aspects of the phenomenon of switching that are not fully understood in both fields.

## Data Availability

The original contributions presented in the study are included in the article/supplementary material; further inquiries can be directed to the corresponding authors.
